# Dynamic effects of thermal acclimation on chytridiomycosis infection intensity and transmission potential in *Xenopus laevis*

**DOI:** 10.1098/rsos.240789

**Published:** 2024-09-11

**Authors:** James E. Noelker, Vitoria Abreu Ruozzi, Kyle D. Spengler, Hunter M. Craig, Thomas R. Raffel

**Affiliations:** ^1^ Department of Biological Sciences, Oakland University, Rochester, MI, USA

**Keywords:** amphibian decline, temperature variability hypothesis, chytridiomycosis, transmission potential, climate disease interactions

## Abstract

The pandemic amphibian pathogen *Batrachochytrium dendrobatidis* (Bd) can cause more severe infections with variable temperatures owing to delays in host thermal acclimation following temperature shifts. However, little is known about the timing of these acclimation effects or their consequences for Bd transmission. We measured how thermal acclimation affects Bd infection in *Xenopus laevis*, using a timing-of-exposure treatment to investigate acclimation effect persistence following a temperature shift. Consistent with a delay in host acclimation, warm-acclimated frogs exposed to Bd immediately following a temperature decrease (day 0) developed higher infection intensities than frogs already acclimated to the cool temperature. This acclimation effect was surprisingly persistent (five weeks). Acclimation did not affect infection intensity when Bd exposure occurred one week after the temperature shift, indicating that frogs fully acclimated to new temperatures within 7 days. This suggests that acclimation effect persistence beyond one week post-exposure was caused by carry-over from initially high infection loads, rather than an extended delay in host acclimation. In a second experiment, we replicated the persistent thermal acclimation effects on Bd infection but found no acclimation effects on zoospore production. This suggests that variable temperatures consistently exacerbate individual Bd infection but may not necessarily increase Bd transmission.

## 1. Introduction

A global reduction of amphibian populations has been partially attributed to the emergence of chytridiomycosis, a fungal skin disease caused by *Batrachochytrium dendrobatidis* (Bd) and the related species *Batrachochytrium salamandrivorans* [1–6]. Like many diseases of ectothermic hosts, chytridiomycosis is strongly temperature-dependent, with most amphibian species becoming more infected at cooler temperatures ([7–9] but see Cohen *et al*. [10]). In addition to these mean-temperature effects on Bd infection, some amphibian species have been found to exhibit periods of increased susceptibility following a sudden temperature shift, owing to delays in host acclimation to the new temperature [8,9]. As climate variability increases and becomes less predictable under the threat of climate change, it is ever more important to understand how temperature and host thermal acclimation responses affect Bd infection and transmission dynamics [9,11].

The temperature variability hypothesis (TVH) of Rohr & Raffel [12] postulates that intra-annual temperature variability increases susceptibility of ectotherm hosts to infectious diseases, owing to delays in host thermal acclimation following unanticipated shifts in temperature. This hypothesis assumes that: (i) host acclimation increases resistance to infection following extended exposure to a new temperature (i.e. ‘beneficial acclimation’; [8]) and (ii) parasites acclimate to new temperatures more rapidly than their hosts [9]. A shorter timescale for parasite acclimation is predicted by metabolic theory because of much higher metabolic rates and shorter process time in smaller organisms [13,14]. Beneficial acclimation effects are commonly observed for measures of thermal tolerance (e.g. critical thermal maximum or CT_max_; [15]); however, other types of thermal acclimation such as ‘cooler is better’ or ‘optimal temperature’ responses are commonly observed when measuring aspects of thermal performance within an organism’s normal temperature range [16]. Beneficial acclimation effects on susceptibility to Bd infection have been observed in adult Cuban treefrogs (*Osteopilus septentrionalis*) and juvenile red-spotted newts (*Notophthalmus viridescens*), consistent with the TVH [8,9]; however, studies in other amphibian species found no acclimation effects or evidence contradicting beneficial acclimation [17–19]. Additional measurements for more host species and life stages would help to determine if there are general patterns in acclimation effects on host resistance to infection. Here, we add to the taxonomic and geographical diversity of species investigated by measuring effects of temperature and thermal acclimation on Bd infection in *Xenopus laevis*.

If delays in host thermal acclimation influence susceptibility to infection, then it is important to determine the timescale over which these acclimation responses occur. One approach to studying the timescale of acclimation responses is to measure how long it takes for effects of past (acclimation) temperatures to dissipate, following an experimental shift to a new temperature. Full acclimation of amphibian thermal traits like CT_max_ can occur on timescales of hours to days, depending on the species examined and the direction of a temperature shift (typically longer following a temperature decrease) [20–23]. However, changes in ectotherm immune parameters, such as complement activity or blood leukocyte profiles, might sometimes occur on longer timescales of days to weeks [24–26]. Prior studies found significant acclimation effects on Bd infections up to two weeks after amphibians were exposed to infection at new performance temperatures, but these effects dissipated and became non-significant at later time points [8,9,18]. These results seem to indicate that it took at least two weeks for many of these host species to fully acclimate their parasite resistance mechanisms to a new temperature; however, it is possible that host acclimation occurred on a shorter timescale. Even if the delay in host acclimation is brief (e.g. <1 week), this short window of parasite opportunity could result in significant differences in infection intensity at later time points. This is because infection is a dynamic process, such that early infection states can have carry-over effects on infection at later time points [27]. It is especially plausible that severe early infections might be self-perpetuating for pathogens like Bd that can suppress host immune responses [28,29]. In the current study, we sought to distinguish between the timing of host acclimation (i.e. a slow-acclimation hypothesis) and the presence of carry-over effects (i.e. a carry-over effect hypothesis) by experimentally varying the timing-of-exposure relative to the timing of a temperature shift. We hypothesized that acclimation of *X. laevis* resistance to infection occurs on relatively short timescale (i.e. < 1 week), but that acclimation effects on Bd infection would still be detectable at later time points owing to carry-over effects of early infection dynamics.

Another important question about how host thermal acclimation affects Bd infection is whether and how individual-level acclimation effects translate into population-level transmission dynamics. We know thermal acclimation status can influence subsequent development of Bd infection on an individual host, at least in terms of Bd load as measured via skin swab quantitative polymerase chain reaction (qPCR; [8,9,17]). However, it is unclear to what extent gene copies on a skin swab represent a host’s potential to transmit the infection to other hosts, for multiple reasons. First, skin swabs only collect DNA from a subsample of a frog’s skin surface and might not accurately represent the true infection burden [30]. Second, qPCR skin swab assays quantify Bd DNA from any source including immature zoosporangia and motile infectious stages (zoospores) left over from experimental inoculations [31–33], contrary to a common assumption that chytrid skin swab assays only measure zoospores released from the host’s skin by mature zoosporangia [30]. Zoospores move via flagellar or amoeboid motility [4] and can survive for multiple days in water [34,35]. At cooler temperatures (4–14.5°C), low exponential death rates (approx. 0.003 h^−1^ [35]) might result in up to 40% zoospore survivorship one week post-inoculation, though the vast majority of zoospores should have encysted by this time point [35]. Once they locate a host, zoospores adhere to and penetrate into the host’s skin, where they develop into mature zoosporangia capable of producing zoospores [4,36]. However, at 10°C, this process can take 6–13 days in frogs like *X. laevis* [35], during which time many immature zoosporangia remain on or close to the skin surface [4,32]. This delay means that at any given time point, qPCR quantification of Bd DNA from skin swabs represents some combination of immature zoosporangia and live or dead zoospores from prior time points, in addition to zoospores released from mature zoosporangia. These various sources of genetic material make it unclear whether Bd DNA detected via skin swabs accurately reflects the release of infective zoospores [31,32] and thus potential for transmission to other hosts [37]. Since prior studies of acclimation effects on Bd infection focused on skin swab qPCR [8,9,18,19], it remains uncertain whether thermal acclimation effects on infection will translate into thermal acclimation effects on transmission potential.

A more direct indicator of transmission potential than qPCR of skin swabs is the rate at which a Bd-infected host releases zoospores into the water column, which can be measured by filtering zoospores out of the water and conducting qPCR on the filter [32,38,39]. Shin *et al*. [39] suggested that filtered water samples might be a superior method for detecting infection than skin swabs because it samples the entire skin surface of the frog. Zoospore production rates have been measured in several species and have tended to positively correlate with skin swab measurements, though the degree of correlation varied among species [39–43]. However, it remains unclear whether Bd load on a swab will always reflect zoospore production, especially early in an experimental infection (i.e. the timescale of thermal acclimation effects observed in prior studies). We hypothesized that zoospore production from Bd-infected *X. laevis* would correlate with Bd load on skin swabs and thus exhibit similar responses to temperature and thermal acclimation treatments.

To address questions about the magnitude and timing of host acclimation effects following a temperature shift (i.e. the slow-acclimation hypothesis versus the carry-over effect hypothesis) and their potential implications for Bd transmission, we performed two controlled-temperature infection experiments with *X. laevis*. To quantify the strength and timing of acclimation effects, we exposed *X. laevis* individuals to infection at different time points before and after a temperature shift and tracked infection levels at weekly intervals using qPCR of skin swabs. To evaluate how acclimation effects influence transmission potential, we quantified zoospore production rates from infected frogs in the second experiment for comparison with swab measurements.

## 2. Methods

### 2.1. Experiment 1A: thermal acclimation effects on *Batrachochytrium dendrobatidis* infection

**Figure 1.**
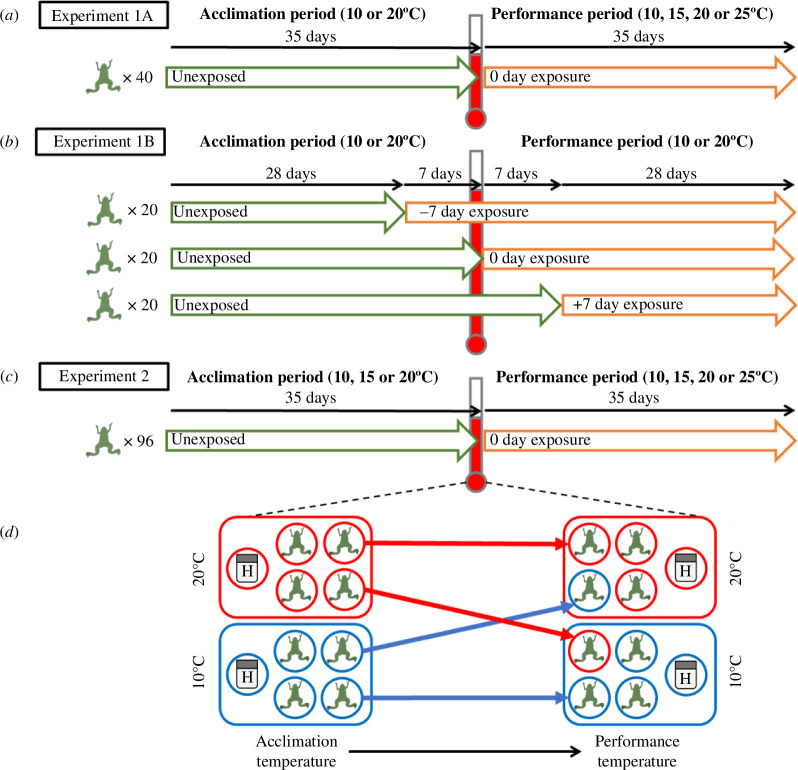
Experimental timelines for (*a*) Experiment 1A, (*b*) Experiment 1B, and (*c*) Experiment 2, summarizing the timing of Bd exposure (green→orange arrows) relative to a temperature shift (thermometer) for each exposure-day treatment. (*d*) Illustration of how frogs assigned to warm (red) or cold (blue) acclimation incubators were all transferred to randomly assigned performance incubators within the same spatial block. Each incubator also contained a ‘blank’ deli cup with a HOBO temperature logger (represented by the icon with ‘H’) and the same water volume as cups housing frogs, to verify temperatures achieved within each incubator.

We investigated the timing of acclimation effects using an individual Bd infection experiment with *X. laevis* in 2021, in which we varied the timing of Bd exposure relative to a shift in temperature. As in prior studies [8,9], the experiment started with a 35 day acclimation period, during which frogs were acclimated to one of two temperatures (10 or 20°C). Frogs were then shifted to new ‘performance’ temperatures (10, 15, 20 or 25°C), followed by a 35 day period at their new constant temperatures (figure 1). The day of the temperature shift was defined as ‘day 0’. To avoid potential for thermal shock, each frog’s container was moved to its new ‘performance’ incubator and allowed to equilibrate to the new temperature over the course of approximately 30 min. Forty ‘zero day exposure’ frogs (20 per acclimation temperature) were inoculated with Bd zoospores on day 0, as soon as frogs had equilibrated to their new temperatures (similar to prior studies; [8,9]). This resulted in a fully crossed 2 × 4 experimental design testing for interactive effects of acclimation temperature (10 or 20°C) and performance temperature (10, 15, 20 or 25°C) on *X. laevis* susceptibility to Bd infection, with five animals per treatment combination which was sufficient to detect hypothesized acclimation effects on Bd infection in a prior study [8]. Unlike in prior studies [8,9], we chose to omit an unexposed (Bd−) control treatment to maximize sample sizes of Bd+ frogs to detect temperature effects on infection dynamics. Uninfected control frogs are essential to draw valid conclusions about how Bd infection affects frog mortality or morbidity [8,9]; however, results from a prior experiment suggested that Bd infection would not cause detectable mortality for individually held *X. laevis* at these experimental temperatures [44]. Bd inoculation procedures are described in a separate section below.

### 2.2. Experiment 1B: varying the day of *Batrachochytrium dendrobatidis* exposure relative to a temperature shift

We ran an additional experiment concurrently with experiment 1A with overlap in temperature treatments. To minimize animal use, 20 of the 40 ‘0 day exposure’ frogs from experiment 1A—those with performance temperatures of 10 or 20°C (10 frogs each)—were also analysed as the ‘0 day’ treatment for experiment 1B. In this experiment, we simultaneously tested for carry-over effects and timing of thermal acclimation effects by adding an ‘exposure day’ treatment, inoculating 20 ‘−7 day’ frogs with Bd zoospores 7 days prior to the temperature shift and inoculating 20 ‘+7 day’ frogs 7 days after the temperature shift (figure 1). Within each of five spatial blocks, four frogs (one for each combination of 2 acclimation × 2 performance temperatures) were exposed to Bd 7 days prior to the temperature shift (−7 exposure day) and four frogs were exposed to Bd 7 days after the temperature shift (+7 exposure day; figure 1). All frogs were shifted to 10 or 20°C performance temperatures on the same day of the experiment (‘day 0’). Combined with the ‘0 day’ frogs from experiment 1A, this resulted in a 2 × 2 × 3 experimental design testing for interactive effects of acclimation temperature (10 or 20°C), performance temperature (10 or 20°C) and timing of exposure relative to the temperature shift (−7, 0 or +7 days). We conducted the entire experimental design in five replicated spatial blocks of eight incubators per block (two incubators per temperature). Each block included one frog per treatment combination (16 frogs per block). Frogs were swabbed for quantification of Bd infection once per week throughout the entire experiment (described in a separate section below).

### 2.3. Experiment 2: temperature dependence of *Batrachochytrium dendrobatidis* load versus zoospore production

We conducted a second individual infection experiment with *X. laevis* in 2022, to evaluate the replicability of acclimation effects observed in experiment 1 and investigate temperature effects on rates of zoospore production. We followed similar procedures to the first experiment by adding an intermediate temperature treatment to make it possible to test for curvilinearity of acclimation temperature effects. Similar to experiment 1, frogs (*n* = 96) were housed at one of three acclimation temperatures (10, 15 or 20°C) for 35 days, shifted to one of four performance temperatures (10, 15, 20 or 25°C), exposed to Bd infection, and held at these new temperatures for another 35 days. Experiment 2 had no timing-of-exposure treatment (i.e. all animals were exposed on the day of the temperature shift, ‘day 0’). At 7 days post-exposure, we briefly placed each frog in clean water prior to swabbing to collect zoospores released into the water (described in a separate section below). For a subset of frogs (10 or 20°C acclimation), we repeated this zoospore-collection procedure at 35 days post-exposure.

### 2.4. Frog maintenance and handling procedures

As soon as frogs were received from the source company (wild-type, random sex, post-metamorphosis snout-vent length to 3.81 cm, Xenopus 1 Corp. Dexter, MI), we treated them for potential pre-existing Bd infection with a combination of a 7 day high-temperature incubation (30°C) and a soak in 0.005% itraconazole solution in amphibian Ringer’s solution for 5 min on each for six consecutive days [45]. Prior to the start of each experiment, frogs were maintained in plastic bins with 4 l of dechlorinated and continuously aerated tap water in our animal care room, which is maintained at 18−20°C on a 12 L : 12 D cycle. Frogs used in experiment 1 had an average initial mass of 6.07 ± 0.17 g, while frogs used in experiment 2 had an average initial mass of 9.17 ± 0.20 g (mean ± s.e.). Frogs exhibiting signs of severe Bd pathology including loss of righting reflex [6] were removed from the study and euthanized via benzocaine overdose (0.5% solution in water) according to our Institutional Animal Care and Use Committee (IACUC) protocol.

During Bd infection experiments, frogs were housed in 1 l deli cups with ventilated lids and 500 ml of dechlorinated tap water (using Kordon AmQuel following manufacturer recommendations; Kordon, LLC, Hayward, CA), placed within custom built Styrofoam incubators with transparent lids to let in room light (12 L : 12 D cycle). Incubators were constructed as described by McWhinnie *et al*. [46]. Frog water was not bubbled during each experiment, but preliminary experiments indicated that dissolved oxygen remained above 50% between water changes. Once per week, frogs received a water change and were fed 3−4 g of live black worms (*Lumbriculus variegatus*). To ensure consistency of water temperatures throughout the experiment, source water was brought to the target experimental temperature prior to each water change. All experiments and animals were used following IACUC protocol 18 011 and IBC protocol 2759–13.

### 2.5. *Batrachochytrium dendrobatidis* inoculation procedure

To prevent possible adaptation to culture conditions, we cultured Bd from cryopreserved culture stocks (stored at −80°C; [47]) less than one month prior to each experiment (strain JEL423 isolated in 2004 from *Peltophryne lemur* in Guabal, Panama [47,48]). We grew initial cultures in 1% tryptone broth at room temperature (approx. 18°C) until there was significant visible growth (approx. 14 days) and then spread 1 ml of the culture broth onto 100 mm 1% tryptone agar plates. Plates were then grown for 10–14 days at room temperature with staggered start dates, starting 8–10 plates per day to ensure we had enough plates producing viable zoospores to generate sufficient inoculate on each scheduled Bd exposure day. To collect zoospores, we pipetted 1 ml of artificial spring water (ASW) onto the agar surface and spread the water around by tilting the plate back and forth for 1–2 min. Zoospores were quantified using a haemocytometer and the inoculate was diluted with ASW to 10^6^ zoospores ml^−1^. We inoculated frogs with Bd after a water change at the acclimation temperature. We moved each frog and its new housing water to the new performance temperature, waited 3 h to allow the water to equilibrate to the new temperature, held the frog over its housing water with a vinyl-gloved hand, and pipetted 1 ml of inoculate (10^6^ zoospores) over the frogs’ dorsal side, allowing excess inoculate to drip into the water.

### 2.6. Swabbing procedure

To quantify levels of Bd infection on each animal throughout the experiment, we swabbed the animals once per week just prior to each water change. For each swabbing session, one researcher held the frog, and a second researcher brushed a fine-tip rayon urethra swab (MW113, Medical Wire & Equipment Company) five times along the ventral surface of each thigh and foot. Animal handlers wore cotton gloves over nitrile gloves to improve their grip when handling frogs [49]. Cotton gloves were removed after handling each individual frog and sanitized for later reuse by washing with quaternary disinfectant and bleach [49]. Within a spatial block (approx. 14 frogs), nitrile gloves were sanitized with 70% ethanol, dried with a paper towel and covered with a fresh pair of cotton gloves before handling the next frog. This procedure effectively prevents cross-contamination by Bd DNA while greatly reducing glove waste [49]. Nitrile gloves were removed and replaced before moving on to the next spatial block.

Swabs were frozen at −20°C for later analysis using a standard qPCR assay as described by Altman & Raffel [17]. To hedge against possible reaction inhibition at 1 : 10 dilution or failure to detect low levels of Bd DNA at 1 : 100 dilution, we ran each swab sample at both levels (one well each).

### 2.7. Quantifying zoospore production (zoospore filtering procedure)

We used methods adapted from DiRenzo *et al*. [40] to quantify the rate of zoospore production by each infected frog in experiment 2. Frogs were placed into a fresh deli cup containing 100 ml of dechlorinated collection water for a target period of 15 min (the precise time was recorded for each frog). The frog was then swabbed and transferred to another new container with 500 ml fresh water (i.e. the regular water change procedure). After removing the frog, we mixed in 100 µl of bovine serum albumin to the zoospore collection water before collecting a 10 ml sample using a 50 ml syringe. We filtered each sample using a 0.45 µm syringe filter (Cytiva Whatman GD/X 13, 6880–1304, 13 mm diameter) and methods adapted from DiRenzo *et al*. [40]. To expedite the filtering process, we used a modified caulk gun fitted with a hydraulic pinch pressure gauge to ensure we never exceeded the syringe filters’ maximum pressure of 75 p.s.i. (517.11 kPa), meaning no more than 51.8 lbs (230.4 N) of force on the syringe plunger (electronic supplementary material, figure S1). Filters were capped, stored and refrigerated for no more than 4 days until extraction. We extracted DNA by adding 200 µl of a 1 : 1 mixture of PrepMan Ultra and 0.25 × TE buffer (diluted 1 × solution TE buffer, Tris-EDTA, pH 8.0, Fisher BioReagents) to the filters using a 1 ml syringe. Filters were then capped and dry incubated at 100°C for 10 min. We then pushed air through the filter with a 1 ml syringe to push the extract out of the filter. The supernatant was collected and stored at –20°C. Samples were later analysed by qPCR assay using the same procedure as for skin swabs (described above).

### 2.8. Replication of temperature treatments

To ensure true replication of temperature treatments, we used an array of 40 incubators for experiment 1 and 44 incubators for experiment 2. Each incubator included a spare deli cup containing 500 ml of tap water that held a HOBO temperature logger (Onset, Bourne, MA) that tracked the temperature throughout the acclimation and performance periods (figure 1). We also checked the calibration of each incubator each day using an aquarium thermometer. Deli cups containing frogs and HOBO loggers were shuffled weekly to control for possible within-incubator variation in temperature.

Frogs were assigned to incubators using a type of split-plot design, similar to that described by Altman *et al*. [50]. Each incubator contained multiple frogs, but each acclimation incubator contained only one frog for a given performance temperature or timing-of-exposure treatment, and each performance incubator contained only one frog from a given acclimation temperature (figure 1). This allowed us to treat incubator as the unit of replication for acclimation and performance temperature treatments (see §2.9).

### 2.9. Statistical analysis

Analyses were conducted using R statistical software (v. 4.1.3 [51]). The number of gene copies detected on each swab or filter was converted to zoospore equivalents (*Z*_e_) using the conversion factor for JEL423 described by Altman & Raffel [17]. The zoospore production rate per 15 min was calculated by dividing each filtered-Bd quantity by the amount of time the frog spent soaking (approx. 15 min), multiplying by 15, and multiplying again by 10 to account for taking a 10 ml subsample out of the 100 ml total collection water volume. To improve normality of model residuals, we log transformed all Bd quantities prior to analysis (ln [Bd *Z*_e_+1]). To analyse Bd infection status, we categorized swab samples as positive if Bd load ≥0.1 *Z*_e_. To test for effects of acclimation temperature, performance temperature, time since exposure and exposure day (for experiment 1B) on log Bd load, we ran linear mixed effects models that included random effects for the acclimation incubator, performance incubator and individual frog identity (function *lmer* in R package *lme4* [52]). We used the *Anova* function from the *car* package to conduct Type II F tests with Kenwood–Rogers degrees of freedom (d.f.; [53]). For each analysis, we collected unstandardized coefficients from models with marginal interaction terms removed, to make sure each coefficient’s sign (+ or −) represented the directionality of effect detected by a Type II test. We also ran versions of these analyses with Bd infection status as a response variable, using the *glmmTMB* function from the *glmmTMB* package [54] to generate generalized linear mixed effects models with binomial error and the same random effects as in the log Bd load models.

To test specific predictions about the relative strength of acclimation effects with the direction or magnitude of a temperature shift, we analysed subsets of the days 7 and 14 data from experiments 1A and 2, with time since exposure as a covariate. To compare the magnitude of acclimation effects for a temperature increase versus a temperature decrease, we quantified differences between 10 and 20°C acclimation treatments for a 10°C performance temperature (20→10 versus 10→10) or a 20°C performance temperature (10→20 versus 20→20). To compare the magnitude of acclimation effects following a larger or smaller temperature shift, we quantified the magnitude of acclimation effects in experiment 2 for a 5°C temperature decrease (15→10 versus 10→10) to compare with a 10°C temperature decrease (20→10 versus 10→10). We made the same comparison for different magnitudes of temperature increase (15→20 versus 20→20 and 10→20 versus 20→20).

To analyse log Bd zoospore production, we ran linear regressions to compare log Bd zoospore production, log Bd load, acclimation temperature and performance temperature. We also used variance partitioning to analyse the amount of shared variance in Bd zoospore production accounted for by both performance temperature and log Bd load (table 1).

**Table 1.** Variable abbreviations and their definitions used for statistical model fitting.

variable	abbreviation	definition
acclimation temperature	AccTemp	the temperature a frog experienced during the (earlier) acclimation period of the experiment (°C).
infection status	BdStatus	a frog’s infection status as either infected (1) or uninfected (0)
day	day	time within the experiment relative to the day of the temperature switch (day 0).
exposure day	DayOfExpo	timing of Bd exposure for each frog, relative to the day of the temperature switch (day 0).
log Bd zoospore production	LnFilterBd.rate	natural log of Bd zoospore equivalents produced over 15 min (ln[Bd Z_e_+1])
log Bd load	LnSwabBd	natural log of Bd zoospore equivalents detected from a swab sample (ln[Bd *Z*_e_+1])
performance temperature	PerfTemp	the temperature a frog was exposed to during the (later) performance period of the experiment (°C).
time since exposure	TimeSinceExposure	number of days elapsed since the timing of Bd exposure for each swab or filter sample

## 3. Results

### 3.1. Temperature and thermal acclimation effects: day 0 exposures (experiments 1A and 2)

**Figure 2.**
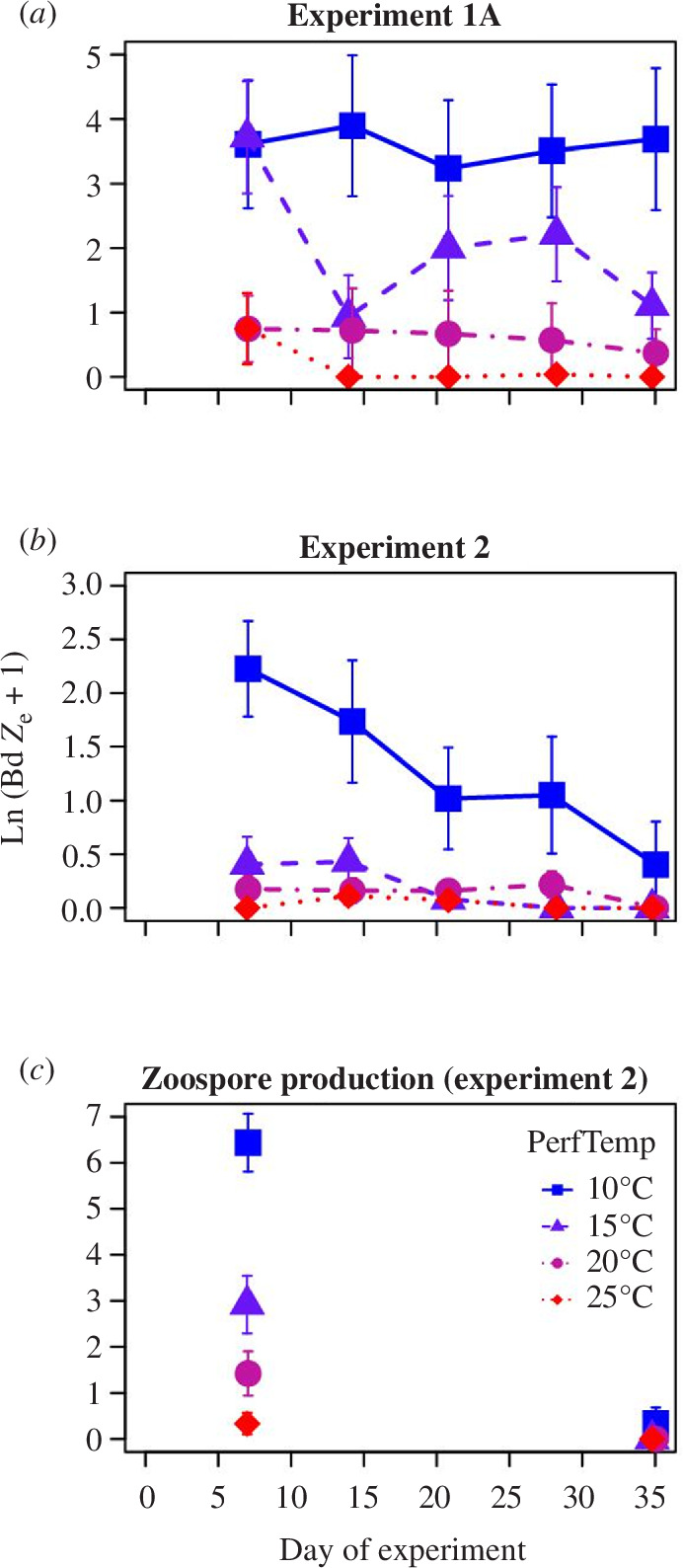
Overall effects of performance temperature on Bd infection dynamics throughout the performance period for ‘day 0’ frogs (i.e. frogs exposed to Bd on the day of the temperature shift). (*a*) Experiment 1A dynamics based on skin swabs (LnSwabBd). (*b*) Experiment 2 dynamics based on skin swabs. (*c*) Zoospore production over 15 min from experiment 2 as measured by water filtration (LnFilterBd.rate). 10°C: blue solid squares and solid lines; 15°C: purple solid triangles and dashed lines; 20°C: solid magenta circles and dot-dashed lines; 25°C: solid red diamonds and dotted lines. Points have been jittered on the *x*-axis. Error bars indicate s.e.

**Figure 3.**
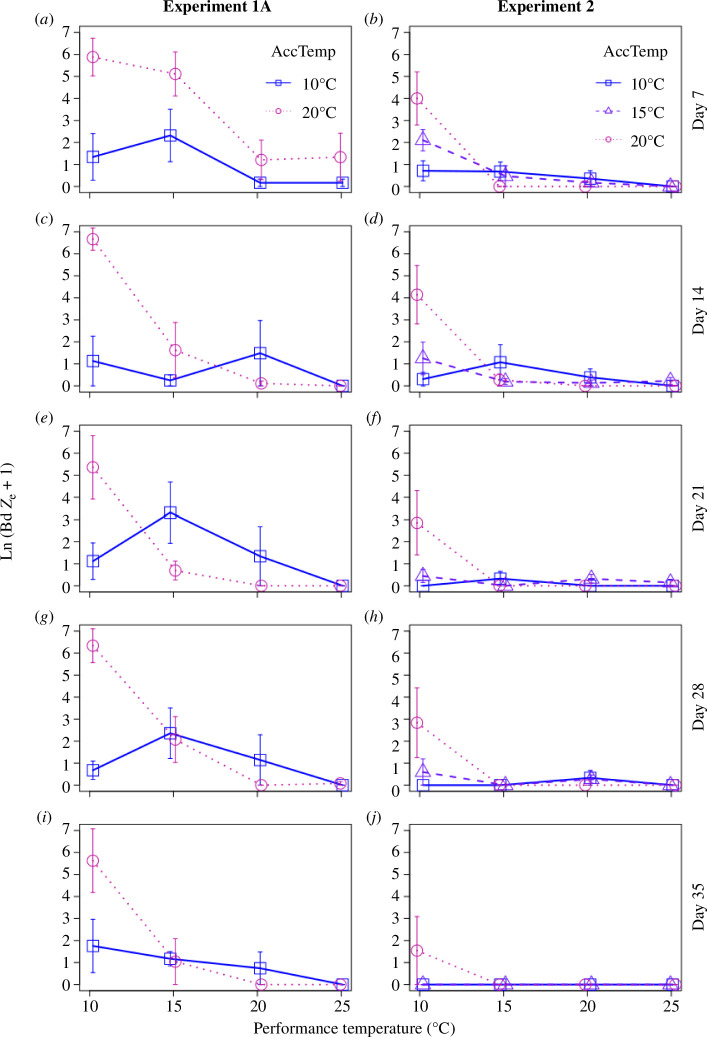
Acclimation and performance temperature effects for: (*a,c,e,g,i*) experiment 1A, and (*b,d,f,h,j*) experiment 2. Acclimation temperatures were 10°C (open blue squares and solid lines), 15°C (open purple triangles and dashed lines) or 20°C (open magenta circles and dotted lines). Each row represents a different week during the performance period of the experiment, with ‘days’ indicating the time since the temperature shift and Bd exposure occurred. Points are jittered on the *x*-axis. Error bars indicate s.e.

Frogs in experiment 1A (2021) became overall more infected than frogs in experiment 2 (2022; figure 3). Nevertheless, there were similar effects of performance and acclimation temperature in both experiments (table 2). Bd infection load significantly decreased with time since exposure in experiment 2 and had a nearly significant decreasing trend in experiment 1A (figures 2 and 3; table 2). There was no mortality during either experiment except for one frog in experiment 2 that was euthanized on day 14 after exhibiting signs of severe Bd-induced pathology (loss of righting reflex [6]). No other frogs exhibited signs of severe Bd pathology (>2 pathology score as described by Voyles *et al*. [6]).

**Table 2.** Interactive effects of performance temperature, acclimation temperature and time since exposure on LnSwabBd, only for frogs exposed to Bd immediately after the temperature shift (‘day 0’ exposure day). (Predictor variables included AccTemp (acclimation temperature), PerfTemp (performance temperature) and TimeSinceExposure (days since Bd exposure). Variable abbreviations are defined in table 1. Statistical tests were based on Kenward–Rogers d.f., which may be non-integers in mixed effects models. Significant results (p < 0.05) are shown in bold.)

experiment	predictor	coefficient	*F-*value	d.f.	*p*
1A	**acclimation temperature**	**0.1142**	**6.9**	**1, 17.1**	**0.017**
	**performance temperature**	**−0.2340**	**33.3**	**1, 29.6**	**<0.001**
	time since exposure	−0.0232	3.8	1, 154.3	0.052
	**AccTemp × PerfTemp**	**−0.0289**	**12.8**	**1, 29.6**	**0.001**
	**AccTemp × TimeSinceExposure**	**−0.0052**	**4.8**	**1, 154.3**	**0.030**
	PerfTemp × TimeSinceExposure	−0.0001	<0.1	1, 154.1	0.947
	AccTemp × PerfTemp × TimeSinceExposure	<0.0001	<0.1	1, 154.1	0.955
2	**acclimation temperature**	**0.0584**	**6.2**	**1, 18.9**	**0.022**
	**performance temperature**	**−0.0736**	**13.4**	**1, 21.3**	**0.001**
	**time since exposure**	**−0.0214**	**23.8**	**1, 380.8**	**<0.001**
	**AccTemp × PerfTemp**	**−0.0174**	**16.9**	**1, 53.0**	**<0.001**
	AccTemp × TimeSinceExpo	−0.0004	0.1	1, 381.9	0.734
	**PerfTemp × TimeSinceExposure**	**0.0039**	**25.0**	**1, 381.5**	**<0.001**
	AccTemp × PerfTemp × TimeSinceExposure	0.0003	2.1	1, 383.6	0.146

There was a highly significant interaction between performance temperature and acclimation temperature in both experiments, in both cases primarily driven by high Bd infection loads in warm-acclimated frogs that were exposed to Bd at cooler performance temperatures (figure 3; table 2). There was also a visible but less-consistent trend towards cool-acclimated frogs having higher Bd loads than warm-acclimated frogs when exposed to Bd at warmer temperatures (figure 3). There were also highly significant negative main effects of performance temperature in both experiments and significant positive main effects of acclimation temperature in both experiments (figure 3; table 2). This main effect of acclimation temperature became reduced with time since exposure (i.e. time since the temperature shift), based on a significant interaction between acclimation temperature and time since exposure in experiment 1A (table 2). The interaction between acclimation temperature and performance temperature was highly significant at day 7 of both experiments and persisted till the end of each experiment, remaining significant on day 35 in experiment 1A and nearly significant in experiment 2 (electronic supplementary material, table S1). We found no curvilinear effects of acclimation temperature with the addition of the 15°C acclimation treatment in experiment 2. Instead, the 15°C group exhibited intermediate Bd loads relative to 10 and 20°C acclimated frogs, indicating a monotonic effect of acclimation temperature on host resistance to Bd infection (figure 3). Binomial mixed effects models revealed similar effects of temperature treatments on Bd infection status but with lower levels of significance (electronic supplementary material, table S3).

### 3.2. Direction and magnitude of temperature shifts

There were stronger acclimation effects on Bd infection intensity following a decrease in temperature from 20 to 10°C (experiment 1A: *F*_1,8_ = 31.4, *p* < 0.001; experiment 2: *F*_1,5_ = 8.5, *p* = 0.033) than for an increase in temperature from 10 to 20°C (experiment 1A: *F*_1,7.6_ < 0.1, *p* = 0.855; experiment 2: *F*_1,5_ = 2.1, *p* = 0.207). In experiment 2, a larger (10°C) decrease in temperature resulted in a significant acclimation effect on Bd infection intensity, whereas a 5°C decrease only resulted in a non-significant trend (20→10: *F*_1,5_ = 8.5, *p* = 0.033; 15→10: *F*_1,11.3_ = 3.3, *p* = 0.097). There were no significant acclimation effects on Bd infection intensity following a temperature increase to a 20°C performance temperature, regardless of the magnitude of the temperature shift (15→20: *F*_1,11_ = 1.2, *p* = 0.299; 10→20: *F*_1,5_ = 2.1, *p* = 0.207).

### 3.3. Timing-of-exposure effects (experiment 1B)

**Figure 4.**
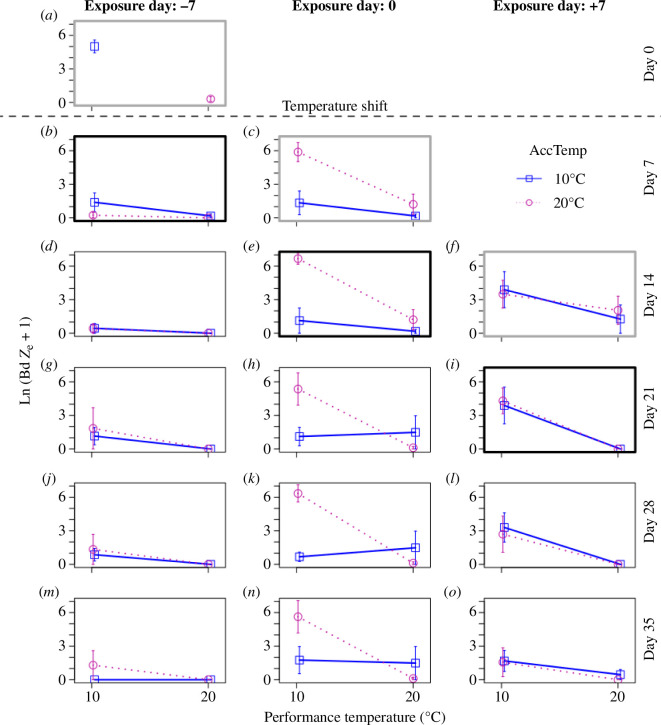
Acclimation effects for timing of exposure treatments from experiment 1B. Panels (*a,b,d,g,j,m*) are frogs that were exposed one week prior to the temperature shift (day −7). Panels (*c,e,h,k,n*) are frogs exposed on the day of the temperature shift (day 0). Panels (*f,i,l,o*) are frogs exposed one week after the temperature shift (day +7). Acclimation temperatures were 10°C (blue squares and solid lines) or 20°C (magenta circles and dotted lines). Plots outlined in thick grey boxes indicate the time point 7 days after Bd exposure. Plots outlined with thick black boxes indicate time points 14 days after Bd exposure. Frogs in panel (*a*) had not yet experienced performance temperature treatments and are therefore shown at their acclimation temperatures. Error bars indicate s.e.

The full analysis of experiment 1A (above) provided a complete picture of how acclimation and performance temperatures influenced Bd infection in the ‘day 0’ frogs for this experiment. For comparison with the day −7 and day +7 treatments, we conducted a follow-up analysis of the subset of day 0 frogs with acclimation or performance temperatures of 10 or 20°C. This analysis was restricted to the first two weeks post-exposure, to maximize the chances of detecting short-term effects of thermal acclimation. We found significant main and interactive effects of acclimation and performance temperatures for this subset of temperature treatments that were consistent with the findings for the full day 0 dataset (figure 4; table 3). There were no significant main or interactive effects of time since exposure in this model, indicating that acclimation and performance temperature effects remained consistent for the first two weeks post-exposure (figure 4; table 3; electronic supplementary material, table S2). No frogs died or exhibited signs of severe Bd pathology during this experiment.

**Table 3.** Interactive effects of performance temperature, acclimation temperature and time since exposure on log Bd load for experiment 1B. (Analysis of temperature effects (PerfTemp & AccTemp) at 7 and 14 days since exposure. Variable abbreviations are defined in table 1. Statistical tests were based on Kenward–Rogers d.f., which may be non-integers in mixed effects models. Note: for day of exposure treatments −7 and +7, only a single frog was housed per incubator for these treatments resulting in no d.f. decimals. Significant results (p < 0.05) are shown in bold.)

day of exposure	predictor	coefficient	*F*-value	d.f.	*p*
−7	**acclimation temperature**	**−0.2679**	**52.1**	**1, 16**	**<0.001**
	performance temperature	0.0456	1.5	1, 16	0.237
	**time since exposure**	**−0.3157**	**99.4**	**1, 16**	**<0.001**
	AccTemp × PerfTemp	−0.0053	0.5	1, 16	0.489
	**AccTemp × TimeSinceExposure**	**0.0576**	**82.8**	**1, 16**	**<0.001**
	**PerfTemp × TimeSinceExposure**	**−0.0337**	**28.4**	**1, 16**	**<0.001**
	**AccTemp × PerfTemp × TimeSinceExposure**	**0.0043**	**11.5**	**1, 16**	**0.004**
0	**acclimation temperature**	**0.2568**	**14.9**	**1, 10.7**	**0.003**
	**performance temperature**	**−0.3143**	**21.3**	**1, 10.6**	**0.001**
	time since exposure	0.0199	<0.1	1, 15.2	0.861
	**AccTemp × PerfTemp**	**−0.0521**	**15.3**	**1, 10.8**	**0.003**
	AccTemp × TimeSinceExposure	−0.0087	0.2	1, 15.5	0.669
	PerfTemp × TimeSinceExposure	−0.0040	0.1	1, 15.5	0.798
	AccTemp × PerfTemp × TimeSinceExposure	−0.0049	1.8	1, 15.7	0.199
+7	acclimation temperature	0.0198	<0.1	1, 16	0.835
	**performance temperature**	**−0.3064**	**10.7**	**1, 16**	**0.005**
	time since exposure	−0.0894	0.7	1, 16	0.405
	AccTemp × PerfTemp	0.0039	<0.1	1, 16	0.837
	AccTemp × TimeSinceExposure	0.0002	<0.1	1, 16	0.993
	PerfTemp × TimeSinceExposure	−0.0294	2.0	1, 16	0.178
	AccTemp × PerfTemp × TimeSinceExposure	−0.0023	0.3	1, 16	0.592

For frogs exposed to Bd on day −7 (i.e. 7 days prior to the temperature shift), there was a highly significant main effect of acclimation temperature on log Bd load, a negative effect of time since exposure and significant interactions with time since exposure for both acclimation temperature and performance temperature (figure 4; table 3). When time points were analysed separately, there was a highly significant negative effect of acclimation temperature at 7 days post-exposure (electronic supplementary material, table S2), at which point, these frogs had not yet experienced their performance temperature treatments (figure 1). By 14 days post-exposure, these frogs had low levels of infection across all treatments and there were no detectable main or interactive effects of either acclimation or performance temperature (electronic supplementary material, table S2). These low levels of infection persisted through the remainder of the experiment (figure 4).

For frogs exposed on day +7, we found highly significant main effects of performance temperature on Bd load during the first two weeks post-exposure but no significant main or interactive effects of acclimation temperature or time since exposure (figure 4; table 3). This performance temperature effect was only significant in the second time point after Bd exposure (electronic supplementary material, table S2), though there was a non-significant trend in the first week towards higher Bd levels at the cooler performance temperature, consistent with the performance temperature effect observed for day 0 frogs (figure 4; electronic supplementary material, table S2). Binomial generalized linear mixed effects models failed to converge when trying to test for timing of exposure effects on Bd infection status.

### 3.4. Zoospore production (experiment 2)

**Figure 5.**
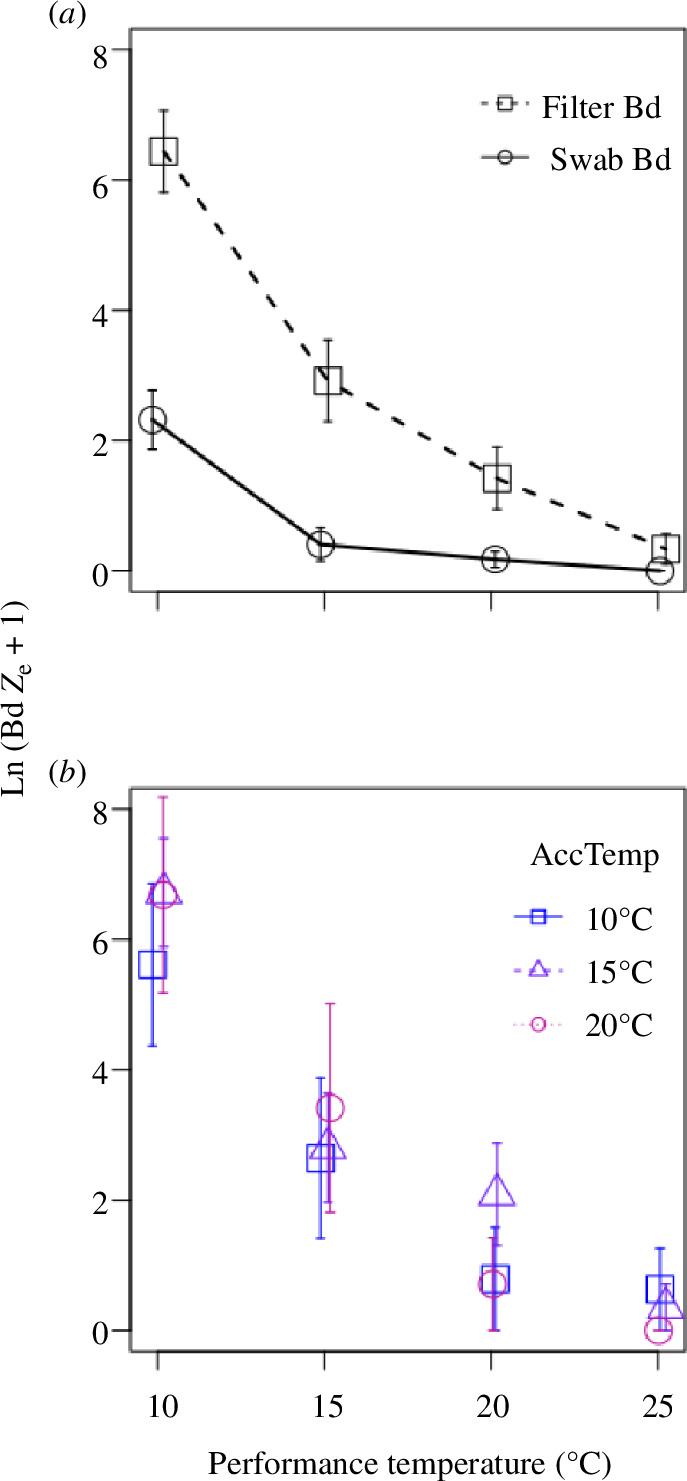
Bd zoospore production over 15 min (LnFilterBd.rate) as a function of performance temperature on day 7 of experiment 2. (*a*) Comparison of zoospore production (open squares and dashed line) to Bd load from skin swabs (LnSwabBd; open circles and solid line). (*b*) Comparison of zoospore production by frogs acclimated to 10°C (open blue squares), 15°C (open purple triangles) or 20°C (open magenta circles). Points are jittered on the *x*-axis. Error bars indicate s.e.

Log Bd zoospore production was significantly higher on day 7 than day 35 (figures 2 and 5), by which time both Bd load (swab samples) and Bd production were almost reduced to zero in this experiment (figures 2 and 4). We therefore focused our analysis of Bd zoospore production on the day 7 time point. Regardless of performance temperature treatment, zoospore production over a 15 min collection period yielded higher Bd quantities in qPCR than standard swab samples (figure 5). This resulted in positive detections of Bd infection even in frogs that tested negative based on swab samples, especially for the warmer temperature treatments (figures 2 and 5).

There was no significant effect of host acclimation temperature on log zoospore production at either time point (day 7: *F*_7,88_ < 0.1, *p* = 0.834; day 35: *F*_3,41_ = 2.1, *p* = 0.151). As a single predictor (i.e. simple linear regression), log swab Bd had a highly significant positive effect on zoospore production on day 7 post-exposure (*F*_1,94_ = 15.7, *p* < 0.001) and accounted for 14.3% of the variance in log zoospore production. Performance temperature was an even more significant positive predictor of zoospore production (*F*_1,94_ = 70.2, *p* < 0.0001), explaining 42.7% of the variance in log zoospore production. Variance partitioning revealed that these two correlated variables explained much of the same variation in zoospore production, with performance temperature explaining 98.0% of the log swab Bd effect, whereas log swab Bd only explained 32.7% of the performance temperature effect. In multiple regression with both predictors, performance temperature continued to have a highly significant negative effect on zoospore production (*F*_1,93_ = 47.0, *p* < 0.0001) but log swab Bd became non-significant (*F*_1,93_ = 0.5, *p* = 0.491).

## 4. Discussion

Both experiments revealed negative effects of current (performance) temperature on Bd infection in *X. laevis* and reduced Bd loads over time, consistent with temperature dependence of Bd infection observed in other species [8,9,18,55,56] and a prior study showing that *X. laevis* mounts an acquired immune response to Bd infection [57]. There was also consistently strong evidence for beneficial acclimation effects on host resistance to Bd infection, as revealed by an interactive effect of acclimation and performance temperatures on frogs exposed to Bd on the day of the temperature shift. In both experiments, cool-acclimated frogs were more resistant to Bd infection at cooler performance temperatures than warm-acclimated frogs, and this effect was stronger following a greater decrease in temperature. Our finding of greater acclimation effects on Bd infection following a temperature decrease than for a temperature increase was similar to results from prior studies of *O. septentrionalis* and *N. viridescens* [8,9]. This provides further support for the TVH of Rohr & Raffel [12], adding to a growing list of species found to have increased susceptibility to Bd infection following a recent drop in temperature [8,9]. As with these other species, *X. laevis* may benefit from experiencing lower levels of individual Bd infection through time if intra-annual temperature shifts occur slowly or infrequently enough to allow frogs to remain fully acclimated to current temperatures for a greater fraction of each year. However, zoospore production measurements from experiment 2 suggest that temperature variability effects on individual infection intensities may have less impact on Bd transmission dynamics than we previously assumed, given our inability to detect thermal acclimation effects on zoospore production (discussed further below).

The strong effects of frog acclimation status on Bd infection persisted for at least five weeks following the temperature shift (and Bd exposure) in both experiment 1A and experiment 2, in contrast to prior studies where acclimation effects on Bd infection were detectable two weeks post-exposure (also two weeks following the temperature shift) but had dissipated by four weeks post-exposure [9,18]. We used a timing-of-exposure treatment to help distinguish between two alternative hypotheses to account for acclimation effect persistence: the slow-acclimation hypothesis and the carry-over effect hypothesis. Contrary to the slow-acclimation hypothesis, which postulates extended delays in the acclimation of frog immune systems to a new performance temperature, frogs exposed to Bd one week following a temperature shift (day +7) exhibited no acclimation effects on Bd infection. This suggests that the frogs became fully acclimated to their new temperatures within the 7 days following the temperature shift, at least in terms of their ability to fight off Bd infection. A thermal acclimation time of less than 7 days for resistance to Bd infection is consistent with measurements of thermal acclimation times for other aspects of amphibian thermal performance (e.g. CT_max_), which have usually been in the range of 1−11 days [58–60].

The lack of acclimation effects in day +7 frogs is, however, consistent with the carry-over effect hypothesis. This finding suggests that the delay in *X. laevis* acclimation following a temperature shift is relatively brief (i.e. less than 7 days), but that Bd can take advantage of this brief window of opportunity to establish higher infection intensities in warm-acclimated frogs that then carry over into later time points (figure 4). Our results suggest that this carry-over effect is strong enough to result in persistently higher infection levels for at least four weeks after frogs become fully acclimated to the new temperature. Infection is a dynamic process, and it is hardly surprising that current Bd infection intensities should be affected by Bd infection intensities in the recent past. However, effects of past events (e.g. exposure to a past acclimation temperature) are typically assumed to dissipate through time, and four weeks is longer than it took in past studies for acclimation effects on Bd infection dynamics to dissipate [8,9]. One way that early acclimation effects on infection dynamics could persist for more than four weeks is if high Bd infection intensities are self-perpetuating in *X. laevis*, perhaps owing to Bd suppression of immune responses [61]. Further research would be needed to confirm that Bd immune suppression is responsible for these effects and under what circumstances carry-over effects result in persistently high infections.

The carry-over hypothesis also predicts that high infection levels prior to a temperature shift should carry over to high infection levels following the shift, regardless of the performance temperature treatment. However, we did not find evidence for this in frogs exposed to Bd infection 7 days prior to a temperature shift (day −7). When these frogs were exposed to Bd at the cooler acclimation temperature, they developed higher infection intensities by the time of the temperature shift (day 0), but these higher infection intensities did not carry over to the next time point (day 7). The low levels of Bd infection by day 7 can be partly explained based on the strong negative effect of warm performance temperatures, given that three of the temperature treatments (warm–warm, warm–cold, cold–warm) involved at least one week of exposure to 20°C. Nevertheless, the lack of persistent high infections in five frogs that experienced the cold–cold treatment is inconsistent with the carry-over effect hypothesis. A second inconsistency is that cold-acclimated frogs exposed to Bd on day 0 did not get heavily infected when exposure occurred at the cold performance temperature, unlike frogs exposed to ostensibly identical (cold–cold) temperature conditions after Bd exposure on day −7 (figure 4). The reason for this inconsistency is unclear, though it may simply highlight the importance of random variation when dealing with small sample sizes (five animals per treatment combination). Another possible difference is that the day −7 and day 0 exposure frogs were exposed to separately prepared Bd inoculates. We made every effort to maintain consistent inoculate quality by standardizing culture methods and concentrations of live (moving) zoospores, but we cannot rule out the possibility that random effects of inoculate quality might have resulted in different levels of infection. Random effects on inoculate quality might also account for differences in overall infection levels between experiments 1 and 2, though this could also be owing to other random effects, such as working with *X. laevis* juveniles with different size distributions.

Zoospore production data from experiment 2 (‘filter Bd’) provided us with an opportunity to evaluate how an aquatic frog’s infection intensity, as measured via DNA quantification of skin swabs, relates to their transmission potential. Our findings were consistent with prior studies of terrestrial frogs that observed higher detectability of Bd zoospores from filtered-water samples than from swab samples, but with a positive correlation between filter Bd and swab Bd [39–43]. However, we failed to detect an effect of host acclimation temperature on Bd zoospore production, despite detecting a significant acclimation effect on Bd skin swabs. These conflicting results suggest the possibility these two response variables are biologically distinct from each other, such that production of Bd zoospores might not always be directly proportional to Bd infection intensity. This is contrary to a common assumption of disease models [62]. A result reinforcing this conclusion was that the negative effect of performance temperature on filter Bd remained significant after controlling for its relationship with swab Bd load, owing to frogs at cool temperatures having high zoospore production relative to their swab Bd loads. The latter result may suggest that individual zoosporangia have higher zoospore production rates at cooler temperatures, consistent with high per-sporangium zoospore production rates in culture reported by Woodhams *et al*. [35]. However, this does not account for the lack of acclimation effects on Bd zoospore production in our experiment. Another plausible reason why Swab Bd and Filter Bd might generate different patterns is if temperature has different effects on development or survival of immature zoosporangia than on zoospore production by mature zoosporangia. In this case, acclimation temperature might have different effects on swab Bd versus filter Bd, especially in the first several days following an experimental infection, assuming that immature zoosporangia make a significant DNA contribution to Bd swab measurements. This assumption seems plausible given that immature zoosporangia remain on or near the epidermal surface of a frog’s skin for multiple days following Bd exposure [32].

The mechanisms driving different acclimation effects on swab Bd versus zoospore production may be unclear, but the implications of these differences could be consequential. Prior studies of host acclimation effects on Bd infection focused solely on swab Bd, a common measure of individual infection intensity that correlates with individual health outcomes (e.g. probability of death; [63]). However, zoospore production should provide a better indication than swab Bd of a host’s ability to transmit Bd to other individuals. Our results do not call into question the individual-level consequences of delays in host acclimation, as postulated by the TVH, but they do warrant caution in extrapolating these results to variable-temperature effects on population-level transmission dynamics. If acclimation effects only influence individual infection intensity, without subsequent effects on transmission to other animals, then the overall effect of variable temperatures on population level disease dynamics might be less important than suggested by prior studies. One caveat is that our zoospore production results focused on a single time point in experiment 2 (7 days post-exposure), and it is possible that the persistent acclimation effects observed in experiment 1A might have led to unobserved acclimation effects on zoospore production at later time points. Nevertheless, our findings demonstrate the importance of examining multiple aspects of Bd infection (e.g. swab Bd and filter Bd) when considering effects of environmental factors on both individual and population level disease processes.

## Data Availability

Data and R code are available here [64]. Supplementary material is available online [65].
